# Salidroside-Mediated Autophagic Targeting of Active Src and Caveolin-1 Suppresses Low-Density Lipoprotein Transcytosis across Endothelial Cells

**DOI:** 10.1155/2020/9595036

**Published:** 2020-06-23

**Authors:** Xiangli Bai, Xiong Jia, Yajing Lu, Lin Zhu, Ying Zhao, Wenzhuo Cheng, Meng Shu, Si Jin

**Affiliations:** ^1^Department of Endocrinology, Institute of Geriatric Medicine, Liyuan Hospital, Tongji Medical College, Huazhong University of Science and Technology, Wuhan, Hubei, China 430077; ^2^Department of Laboratory Medicine, Liyuan Hospital, Tongji Medical College, Huazhong University of Science and Technology, Wuhan, Hubei, China 430077

## Abstract

Subendothelial retention of apolipoprotein B100-containing lipoprotein, such as low-density lipoprotein (LDL), is the initial step of atherogenesis. Activation of autophagy exhibits beneficial effects for the treatment of atherosclerosis. In our previous study, we demonstrated that hyperglycemia suppressed autophagic degradation of caveolin-1, which in turn resulted in acceleration of caveolae-mediated LDL transcytosis across endothelial cells and lipid retention. Therefore, targeting the crossed pathway in autophagy activation and LDL transcytosis interruption may be a promising antiatherosclerotic strategy. In metabolic diseases, including atherosclerosis, salidroside, a phenylpropanoid glycoside compound (3,5-dimethoxyphenyl) methyl-*β*-glucopyranoside), is the most important compound responsible for the therapeutic activities of *Rhodiola*. However, whether salidroside suppresses LDL transcytosis to alleviate atherosclerosis has not yet been elucidated. In the present study, we demonstrated that salidroside significantly decreased LDL transcytosis across endothelial cells. Salidroside-induced effects were dramatically blocked by AMPK (adenosine monophosphate-activated protein kinase) inhibitor (compound c, *AMPKα* siRNA) and by overexpression of exogenous tyrosine-phosphorylated caveolin-1 using transfected cells with phosphomimicking caveolin-1 on tyrosine 14 mutant plasmids (Y14D). Furthermore, we observed that salidroside promoted autophagosome formation via activating AMPK. Meanwhile, the interaction between caveolin-1 and LC3B-II, as well as the interaction between active Src (indicated by the phosphorylation of Src on tyrosine 416) and LC3B-II, was significantly increased, upon stimulation with salidroside. In addition, both bafilomycin A_1_ (a lysosome inhibitor) and an AMPK inhibitor (compound c) markedly prevented salidroside-induced autophagic degradation of p-Src and caveolin-1. Moreover, the phosphorylation of caveolin-1 on tyrosine 14 was disrupted due to the downregulation of p-Src and caveolin-1, thereby directly decreasing LDL transcytosis by attenuating the number of caveolae on the cell membrane and by preventing caveolae-mediated LDL endocytosis released from the cell membrane. In ApoE^−/−^ mice, salidroside significantly delayed the formation of atherosclerotic lesions. Meanwhile, a significant increase in LC3B, accompanied by attenuated accumulation of the autophagy substrate SQSTM1, was observed in aortic endothelium of ApoE^−/−^ mice. Taken together, our findings demonstrated that salidroside protected against atherosclerosis by inhibiting LDL transcytosis through enhancing the autophagic degradation of active Src and caveolin-1.

## 1. Introduction

Atherosclerotic cardio- or cerebrovascular diseases are common causes of morbidity and mortality worldwide [1]. Subendothelial retention of apolipoprotein B- (APOB/apoB100-) containing lipoproteins, such as low-density lipoprotein (LDL), is the initial step of atherogenesis [2–4]. The gap between vascular endothelial cells is roughly 3-6 nm in diameter, which only allows water and inorganic salts, and several small molecules, to pass through. However, the diameter of LDL is 20-50 nm; thus, the only way for LDL particles to traffic across the intact endothelial barrier is through a transporting process called transcytosis [3]. In endothelial cells, LDL transcytosis is predominantly mediated by caveolae, which are specialized lipid rafts that form 50-100 nm flask-shaped invaginations in the plasma membrane [5–7].

Caveolin-1 and cavin-1 are two essential and structural components of caveolae and represent small invaginations of the plasma membrane that form lipid vesicles [7]. Caveolin-1 was initially identified as a substrate for c-src tyrosine kinase, which phosphorylates caveolin-1 on tyrosine 14 [8, 9]. Tyrosine-phosphorylated caveolin-1 (p-caveolin-1) can drive caveolae reconformation and subsequent internalization from the cell membrane [10]. Furthermore, tyrosine-phosphorylated caveolin-1 increased the number of caveolae on the cell membrane by promoting the expression of caveolin-1 and cavin-1 via transcriptional regulation of early growth response-1 [11].

Macroautophagy/autophagy is a homeostatic process that occurs in all eukaryotic cells and involves sequestration of cytoplasmic components in double-membraned autophagosomes that subsequently fuse with lysosomes in which their cargo is delivered for degradation and recycling purposes [12].

Autophagy acts as a therapeutic target for preventing and ameliorating atherosclerosis via numerous pathways to protect cells against oxidative stress, inflammation, and apoptosis [13–16]. In our previous study, we demonstrated that high glucose suppressed autophagic targeting of caveolin-1. Therefore, more caveolin-1 was accumulated in the cytosol and utilized to increase the caveolae on the cell membrane to facilitate the transcytosis of LDL across endothelial cells [17].

Salidroside, a phenylpropanoid glycoside compound, has been shown to alleviate metabolic diseases, such as atherosclerosis and diabetes, by modulating various synergistic pathways that control oxidative stress, inflammation, mitochondria, autophagy, and cell death, as well as AMPK signaling [18]. In our previous study, we demonstrated that salidroside effectively alleviated the progression of insulin resistance and atherosclerosis by activating AMPK to suppress reactive oxygen species generation and inflammasome activation [19–21].

In the present study, we demonstrated that treatment with salidroside enhanced the autophagic degradation of active Src and caveolin-1 by activating AMPK. Subsequently, the expression of caveolin-1 and p-caveolin-1 was decreased, thereby resulting in inhibition of LDL transcytosis across endothelial cells to reduce lipid accumulation in the vascular wall.

## 2. Materials and Methods

### 2.1. Primary Cultures of Human Umbilical Vein Endothelial Cells (HUVECs)

HUVECs were isolated as previously described [22]. Cells were routinely cultured in endothelial cell medium (ScienCell, 1001), containing 5% fetal bovine serum (ScienCell, 2500), 100 U·mL^−1^ penicillin, 100 U·mL^−1^ streptomycin (ScienCell, 0503), and 30 *μ*g·mL^−1^ endothelial cell growth supplement (ScienCell, 1052) at 37°C in an incubator with a humidified atmosphere of 5% CO_2_. Cells were used at passages 2-7.

### 2.2. Reagents

The following reagents were used: salidroside (purity *N* 98%) from the National Institute for Food and Drug Control (Beijing), bafilomycin A_1_ (Sigma-Aldrich, 196000), compound c (Selleck, S7840), LDL (Yiyuan Biotechnology, YB-001), FITC (Sigma-Aldrich, 46950), DMSO (Sigma-Aldrich, 34869), and DiI-LDL (Yiyuan Biotechnology, YB-007).

### 2.3. LDL Labeling

LDL and FITC were mixed at a ratio of 6 mg LDL : 1 mmol FITC and incubated at 37°C for 2 h. The mixture was dialyzed against PBS for 72 h at 4°C to remove unbound FITC.

### 2.4. Flow Cytometry of FITC-LDL Uptake Analyses

HUVECs were seeded in 12-well plates (Roche Diagnostics Corporation, 3513) and were incubated with serum-free Opti-MEM (Gibco, 31985-070), containing FITC-LDL (50 *μ*g/mL) for 3 h. Cells were harvested using 0.125% trypsin (without EDTA), and LDL uptake was measured by flow cytometry (Mindry, Bricyte E6). HUVECs treated with naïve LDL were used as a negative control. To obtain the real fluorescence due to FITC-LDL uptake, the background fluorescence was subtracted from the mean FITC-LDL fluorescent intensity of each sample, FSC/FSS scatter diagrams were plotted, and 2000 events were recorded.

### 2.5. Western Blotting Analyses

20 *μ*g of total protein in 10 *μ*L supernatant was loaded and separated by SDS-PAGE gel and immunoblotted with indicated primary antibodies. Membranes were incubated with horseradish peroxidase- (HRP-) conjugated secondary antibody (1 : 10,000; Beyotime, A0208) for 1 h, and the immunoreactive bands were visualized by chemiluminescence. The choice of 20 *μ*g total protein as a proper sampling amount was based on our preliminary experiments of linear range detection, which found that 20 *μ*g protein was within the combined linear range of both target proteins and housekeeping loading control. The following primary antibodies were used at 1 : 1000 dilution: rabbit anti-LC3B (Cell Signaling Technology, 3868), anti-SQSTM1/p62 (Cell Signaling Technology, 39749), anti-caveolin-1 (Cell Signaling Technology, 3267), anti-p-caveolin-1 (Cell Signaling Technology, 3251), anti-actin (Cell Signaling Technology, 4970), anti-AMPK (Cell Signaling Technology, 9158), anti-p-AMPK (Cell Signaling Technology, 50081), anti-p-Src (Cell Signaling Technology, 6943), anti-Src (Cell Signaling Technology, 2109) antibodies, anti-cavin-1 (Proteintech, 18892-1-AP), anti-c-Cbl (Proteintech, 25818-1-AP), and anti-flag (Proteintech, 50543-1-AP). Goat anti-rabbit IgG HCS (Abbkine, A25222), goat anti-rabbit IgG (Abbkine, A21020), and goat anti-mouse IgG (Abbkine, A21010) secondary antibodies were used at 1 : 10,000 dilution.

### 2.6. LDL Transcytosis

The amount of LDL transcytosis was measured according to previous reports [23]. In brief, HUVECs were seeded on polyester transwell membranes (Costar, 3470). The integrity of the cell monolayer was evaluated as previously described [24]. Two inserts containing cell monolayers with equal integrity were assigned to the same group, and two different inserts were considered: the noncompetitive insert and the competitive insert. The competitive insert was treated with FITC-LDL (50 *μ*g·mL^−1^) and sixfold excess of unlabeled LDL to determine paracellular transport, whereas the noncompetitive insert was treated with FITC-LDL (50 *μ*g·mL^−1^) alone to determine the total transport of transendothelial LDL. Samples were collected from the lower chambers and further dialyzed against PBS (Thermo Fisher Scientific,10010031) to remove free FITC. The intensity of FITC was measured by a fluorescence spectrophotometer (Tecan, Ininite F200PRO) with excitation and emission wavelengths of 490 nm and 520 nm, respectively. Background fluorescence of serum-free Opti-MEM (Gibco, 31985-070) was subtracted from the fluorescence of each sample. The amount of LDL transcytosis was calculated by the difference between the fluorescence intensity of the noncompetitive insert and the competitive insert.

### 2.7. Small Interfering RNA (siRNA) Transfection

HUVECs were transfected with indicated siRNA or scrambled siRNA (Guangzhou RiboBio, China, siNO5815122147) using a HiPerFect transfection reagent (Qiagen, 301705) according to the manufacturer's protocol. *AMPKα* siRNA was from Santa Cruz Biotechnology, CA, USA. The sequences are as follows: *Caveolin-1* siRNA sense (5′-CGAGAAGCAAGUGUACGACdTdT-3), *Caveolin-1* siRNA antisense (5′-GUCGUACACUUGCUUCUCGdTdT-3′), *ATG5* siRNA sense (5′-GGAACAUCACAGUACAUUUdTdT-3′), and *ATG5* siRNA antisense (5′-AAAUGUACUGUGAUGUUCCdTdT-3′).

### 2.8. Coimmunoprecipitation Analyses

Cells were lysed on ice in immunoprecipitation cell lysis buffer (Beyotime, P0031), containing a protease inhibitor cocktail (Roche, 049693132001). The lysate was centrifuged at 15,200 × g for 15 min at 4°C, and the supernatant was quantified by BCA (Thermo Fisher Scientific, 23235) analyses. For the coimmunoprecipitation assay, an amount of 500 *μ*g of protein in 250 *μ*L supernatant was incubated overnight at 4°C with anti-c-Cbl (1 : 50), anti-p-Src (1 : 50), anti-LC3B (1 : 50), and anti-caveolin-1 (1 : 50), followed by precipitation with 20 *μ*L of Pierce® Protein A/G Agarose (Thermo Fisher Scientific, 20421) for 2 h at room temperature. Normal rabbit IgG was used as a negative control. The precipitated complexes were separated by SDS-PAGE gel and immunoblotted with anti-c-Cbl, anti-p-Src, anti-caveolin-1, and anti-LC3B. To reduce the signals from the denatured IP antibody, the secondary antibody anti-rabbit IgG light chain specific (Cell Signaling Technology, 93702) was used at 1 : 1000 dilution.

### 2.9. Plasmid Transfection

The GFP-LC3B plasmid was a gift from Professor Ruiguang Zhang (Cancer Center, Union Hospital, Tongji Medical College, Huazhong University of Science and Technology, Wuhan, Hubei, China). Vector plasmid (pcDNA3.1; Tsingke, Y0014778-3), Y14D plasmids (phosphomimicking caveolin-1 on tyrosine 14 and the C-terminal of the plasmids fused with three flags), and Y14F plasmids (phosphodefective caveolin-1 on tyrosine 14 and the C-terminal of the plasmids fused with three flags) were all synthesized by Tsingke, Beijing, China. HUVECs were seeded and transfected with indicated plasmid at a concentration of 1 *μ*g for 24 h using an Effectene transfection reagent (Qiagen, 301427) according to the manufacturer's protocol. HUVECs were infected with GFP-RFP-LC3 lentivirus (Gene, GTCA1431079923QA) according to the manufacturer's instructions. The MOI used for infection is 20. After infection, cells were cultured in endothelial cell medium containing additional 0.25 *μ*g/mL puromycin.

### 2.10. Transmission Electron Microscopy

After indicated treatments, cells were fixed with 2.5% glutaraldehyde in 0.1 M sodium cacodylate buffer and stored at 4°C until embedding. Then, cells were postfixed with 1% OsO_4_ in 0.1 M cacodylate buffer (pH 7.2), containing 0.1% CaCl_2_ for 1 h at 4°C. After rinsing with cold distilled water, cells were dehydrated through a graded series of ethanol (30%–100%). Samples were embedded in EMbed-812 (EMS, 14120), and after polymerization of the resin at 60°C for 36 h, serial sections were cut using an ultramicrotome (Leica, Germany) and mounted on formvar-coated slot grids (EMS, GA300-Cu). Sections were stained with 4% uranyl acetate and lead citrate and examined under a Tecnai G2 F20 S-TWIN transmission electron microscope (FEI, American).

### 2.11. Confocal Imaging Analyses of LDL Uptake

Cells were incubated with DiI-LDL (50 *μ*g/mL) for 24 h and then treated as indicated. Images were obtained with a confocal laser scanning microscope (FV3000; Olympus) using a 40x objective. Fluorescence images were analyzed using ImageJ software (Santa Clara, CA, USA). Individual microscopic fields were randomly selected to include at least 15 cells, and the numbers of cells were counted. The fluorescence intensities were normalized to the number of cells.

### 2.12. Experiments in ApoE^−/−^ Mice

Animals were treated in accordance with the *Guide for the Care and Use of Laboratory Animals* published by the US National Institutes of Health and approved by the local animal care committee. All studies involving animals are reported in accordance with the ARRIVE guidelines for reporting experiments involving animals [25]. Every effort was made to minimize animal suffering and reduce the number of animals used.

12-week-old male ApoE^−/−^ mice were purchased from HFK (Beijing, HFK Bioscience Co., Ltd., Beijing). All animals were maintained in a controlled environment with a light/dark cycle of 12 h, a temperature of 20 ± 2°C, and a humidity of 50 ± 2%. After giving them a week to adapt to this environment, mice were fed a standard diet for 2 weeks and then followed by a “Western diet” (21% fat, 0.15% cholesterol) for 8 weeks. ApoE^−/−^ mice (*n* = 7) were randomly assigned into the vehicle group and the salidroside group. The vehicle group received p.o. with 0.9% saline. The salidroside group received p.o. with salidroside (purity > 98%, National Institute for Food and Drug Control, Beijing, China; 50 mg·kg^−1^·day^−1^; *n* = 7) for 8 weeks and then killed under anesthesia. The ascending aorta was collected for Oil Red O staining. Serial cross-sections (8 *μ*m) of the heart throughout the entire aortic valve area were cut in a cryostat (Leica CM1900), and the atherosclerotic lesions were stained with Oil Red O. Plaque size was quantified using the ImageJ pro plus software as described previously [26, 27]. The sections stained with LC3B (1 : 50; Cell Signaling Technology, 3868) or SQSTM1 (1 : 50; Cell Signaling Technology, 39749) were examined under light microscopy at a magnification of ×400 with a semiquantitative scoring system (0 to 4) by a method described previously [28].

### 2.13. Statistical Analyses

Data are expressed as the mean ± SEM from at least three independent experiments. Individual group statistical comparisons were analyzed by an unpaired Student *t*-test, whereas multiple-group comparisons were evaluated by one-way ANOVA with post hoc testing. *p* < 0.05 was considered statistically significant.

## 3. Results

### 3.1. Salidroside Suppresses LDL Transcytosis

**Figure 1:**
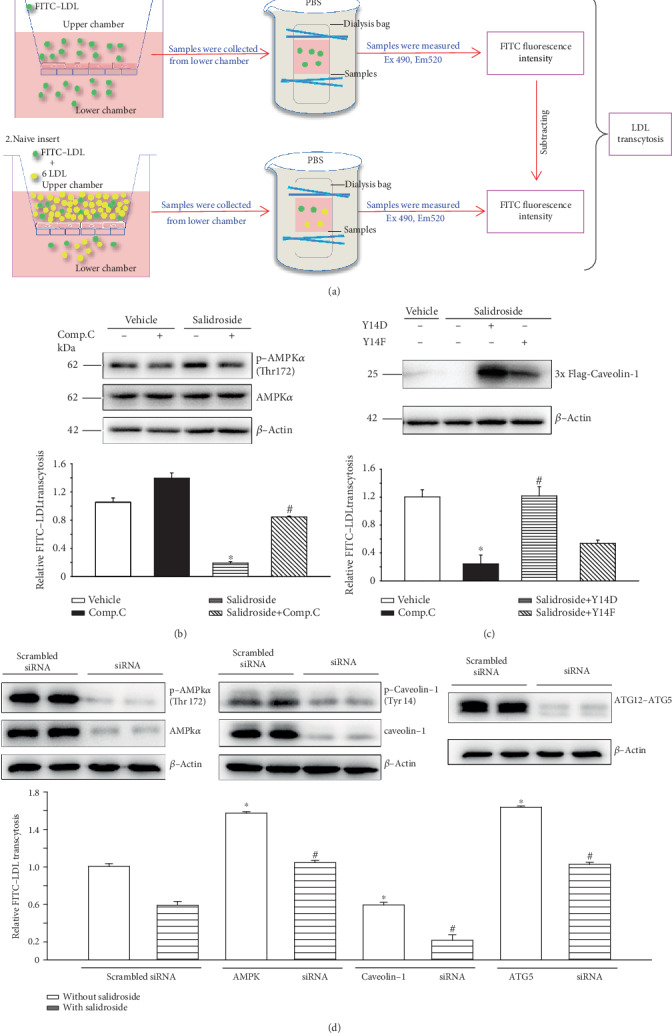
Salidroside suppresses LDL transcytosis. (a) HUVECs were seeded and cultured in a monolayer on a polyester membrane (0.4 *μ*m) placed in the upper chamber of a dual-chamber well. (1) The medium in the upper chamber was added FITC-LDL (50 *μ*g/mL), and samples were collected from the lower chamber and further dialyzed against PBS to remove free FITC. The amount of FITC-LDL was measured in the samples as the amount of total LDL transported (control insert). (2) The medium in the upper chamber contained FITC-LDL (50 *μ*g/mL) and sixfold excess of unlabeled LDL. Similarly, samples from the lower chamber were collected and dialyzed. The amount of FITC-LDL was measured in the samples as the amount of LDL transported paracellularly (naive insert). The amount of LDL transcytosis is the difference of FITC fluorescent intensity subtracted from the control insert to the naive insert. (b) HUVECs were treated with salidroside (1 *μ*M) for 3 h in the presence or absence of compound c (10 *μ*M, 3 h). Upper panel: the representative western blots showing the expression of p-AMPK, AMPK, and *β*-actin. Lower panel: the amount of LDL transcytosis was measured and normalized to that obtained in the vehicle control group. ^∗^*p* < 0.05 versus vehicle. ^#^*p* < 0.05 versus salidroside (*n* = 3). (c) HUVECs were transfected with Y14D (phosphomimicking Y14D caveolin-1 mutant plasmids) or Y14F (a phosphodefective caveolin-1 mutant plasmid) (1 *μ*g, 24 h), followed by exposure to salidroside (1 *μ*M, 3 h). Upper panel: the representative western blots showing the exogenous expression of caveolin-1 mutants (3x flag-caveolin-1) and *β*-actin. Lower panel: the amount of LDL transcytosis was measured and normalized to that obtained in the vehicle control group. (d) HUVECs were transfected with scrambled siRNA (20 nM) or indicated siRNA (20 nM) for 48 h and then treated with salidroside (1 *μ*M, 3 h) or vehicle for 3 h. Upper panel: the representative western blots showing the expression of AMPK*α*, p-AMPK*α*, caveolin-1, p-caveolin-1, ATG12-ATG5, and *β*-actin. Lower panel: the amount of LDL transcytosis was measured and normalized to that obtained in the vehicle control group. ^∗^*p* < 0.05 versus vehicle. ^#^*p* < 0.05 versus salidroside (*n* = 3).

To determine whether salidroside could alter the degree of LDL transport across human vascular endothelial cells (HUVECs), we evaluated the degree of LDL transcytosis across HUVECs using an established nonradioactive *in vitro* approach (Figure 1(a)) [23]. As shown in Figure 1(b), salidroside significantly suppressed LDL transcytosis across the HUVEC monolayer. In addition, compound c recovered salidroside-attenuated LDL transcytosis (Figure 1(b)). Similarly, salidroside-suppressed LDL transcytosis was restored by *AMPKα* siRNA or *ATG5* siRNA transfection (Figure 1(d)). Furthermore, to investigate the role of p-caveolin-1 in salidroside-inhibited LDL transcytosis, two caveolin-1 mutant plasmids were established, including Y14D plasmids (phosphomimicking caveolin-1 on tyrosine 14) and Y14F plasmids (phosphodefective caveolin-1 on tyrosine 14, used as dominant negative p-caveolin-1). Additionally, restoring the expression of p-caveolin-1 by transfecting salidroside-treated cells with Y14D plasmids significantly blocked the downregulation of salidroside-mediated transcytosis of LDL (Figure 1(c)). However, transfecting salidroside-treated cells with Y14F plasmids did not have a significant effect on salidroside-decreased transcytosis of LDL (Figure 1(c)). Furthermore, salidroside-attenuated LDL transcytosis was aggravated by *caveolin-1* siRNA transfection (Figure 1(d)). Collectively, salidroside suppressed LDL transcytosis across endothelial cells.

### 3.2. Decreased LDL Uptake in Salidroside-Treated HUVECs

**Figure 2:**
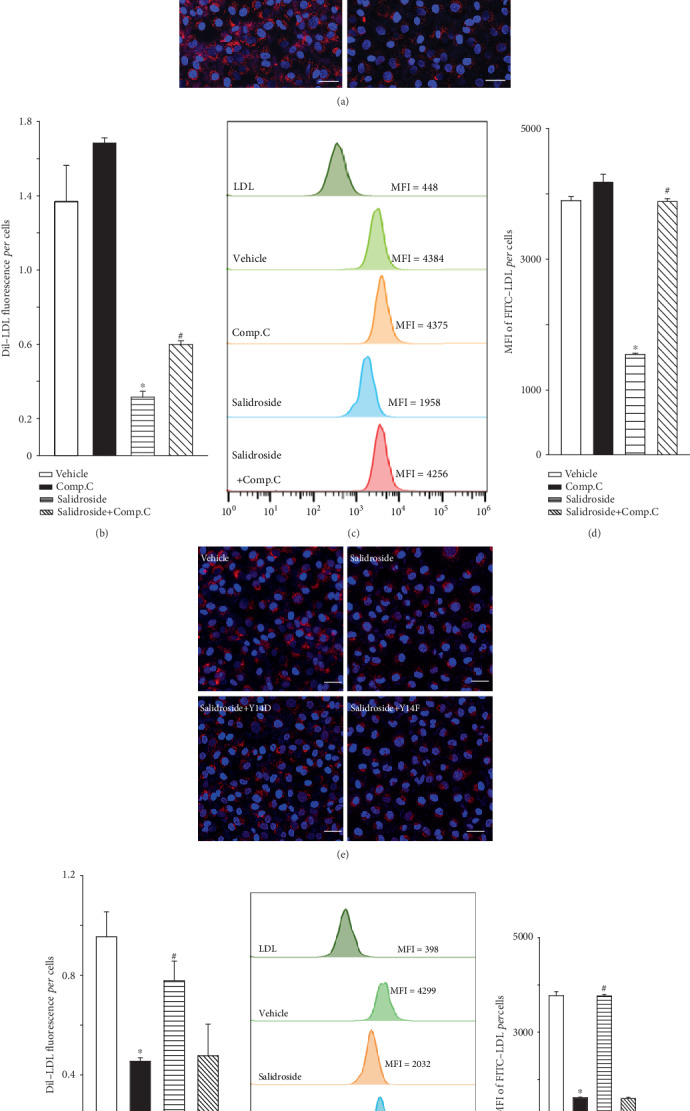
Salidroside inhibits LDL uptake. (a, b) HUVECs were treated with salidroside (1 *μ*M, 3 h) in the presence or absence of compound c (10 *μ*M, 24 h), followed by DiI-LDL (50 *μ*g/mL, 24 h) treatment. (a) Confocal microscopic images of DiI-LDL uptake in HUVECs. The DiI-LDL (red) fluorescence indicates DiI-LDL particle, and the blue fluorescence represents nucleus in HUVECs. Scale bars: 50 *μ*m. (b) Quantification of DiI-LDL uptake represented by DiI-LDL fluorescence in HUVECs. (c, d) HUVECs were treated with salidroside (1 *μ*M, 3 h) in the presence or absence of compound c (10 *μ*M, 3 h) and FITC-LDL (50 *μ*g/mL, 3 h) treatment. Cells were harvested, and the MFI of FITC-LDL in cells was measured by flow cytometry analyses. (c) Representative flow cytometry images show the MFI of HUVECs. (d) Quantification summary of FITC-LDL uptake represented by MFI in HUVECs. (e, f) HUVECs were transfected with Y14D plasmids or Y14F plasmids (1 *μ*g, 24 h), followed by exposure to salidroside (1 *μ*M, 3 h) and DiI-LDL (50 *μ*g/mL, 24 h). (e) Confocal microscopic images of DiI-LDL uptake in HUVECs. The DiI-LDL (red) fluorescence indicates DiI-LDL particles, and the blue fluorescence represents the nucleus in HUVECs. Scale bars: 50 *μ*m. (f) Quantification of DiI-LDL uptake in HUVECs. (g, h) HUVECs were transfected with Y14D plasmids or Y14F plasmids (1 *μ*g, 24 h), followed by exposure to salidroside (1 *μ*M, 3 h) and FITC-LDL (50 *μ*g/mL, 3 h) treatment. Cells were harvested, and the uptake of FITC-LDL was measured by flow cytometry analyses. (g) Representative flow cytometry images show the MFI of HUVECs. (h) Quantification summary of FITC-LDL uptake in HUVECs. ^∗^*p* < 0.05 versus vehicle. ^#^*p* < 0.05 versus salidroside (*n* = 3).

LDL uptake by endothelial cells is an intermediate step of LDL transcytosis [29]. Therefore, the intracellular concentration of LDL in HUVECs reflects the activity of LDL transcytosis. In the present study, confocal imaging was used to determine the uptake of DiI-labeled LDL. Meanwhile, flow cytometry analyses were performed to evaluate FITC-labeled LDL uptake in HUVECs in which the fluorescent intensity of individual cells reflected the extent of LDL uptake.  Figure 2(a) shows that after incubation of HUVECs with DiI-LDL, cells were full of small, individual, and discrete vesicles that were present throughout the cells. Treatment with salidroside significantly diminished the fluorescence intensity of DiI-LDL in HUVECs (Figures 2(a) and 2(b)), thereby indicating a decrease in LDL uptake. Conversely, compound c restored the salidroside-suppressed uptake of DiI-LDL (Figures 2(a) and 2(b)). Similarly, the mean fluorescence intensity (MFI) of FITC-LDL in HUVECs, representing the level of FITC-LDL uptake, was markedly attenuated in salidroside-treated cells (Figures 2(c) and 2(d)). In addition, treatment with compound c almost completely restored the MFI of FITC-LDL uptake in HUVECs upon salidroside treatment (Figures 2(c) and 2(d)). Furthermore, as shown in Figures 2(e) and 2(f), overexpression of exogenous p-caveolin-1 by Y14D plasmid transfection blocked salidroside-suppressed DiI-LDL and FITC-LDL uptake in HUVECs (Figures 2(e)–2(h)). However, Y14F plasmid transfection did not significantly influence salidroside-inhibited LDL uptake (Figures 2(e)–2(h)). Taken together, treatment with salidroside attenuated LDL uptake by activating AMPK and by suppressing caveolin-1 phosphorylation.

### 3.3. Salidroside Activates AMPK and Increases Autophagic Degradation of p-Src and Caveolin-1 in a Dose-Dependent Manner

**Figure 3:**
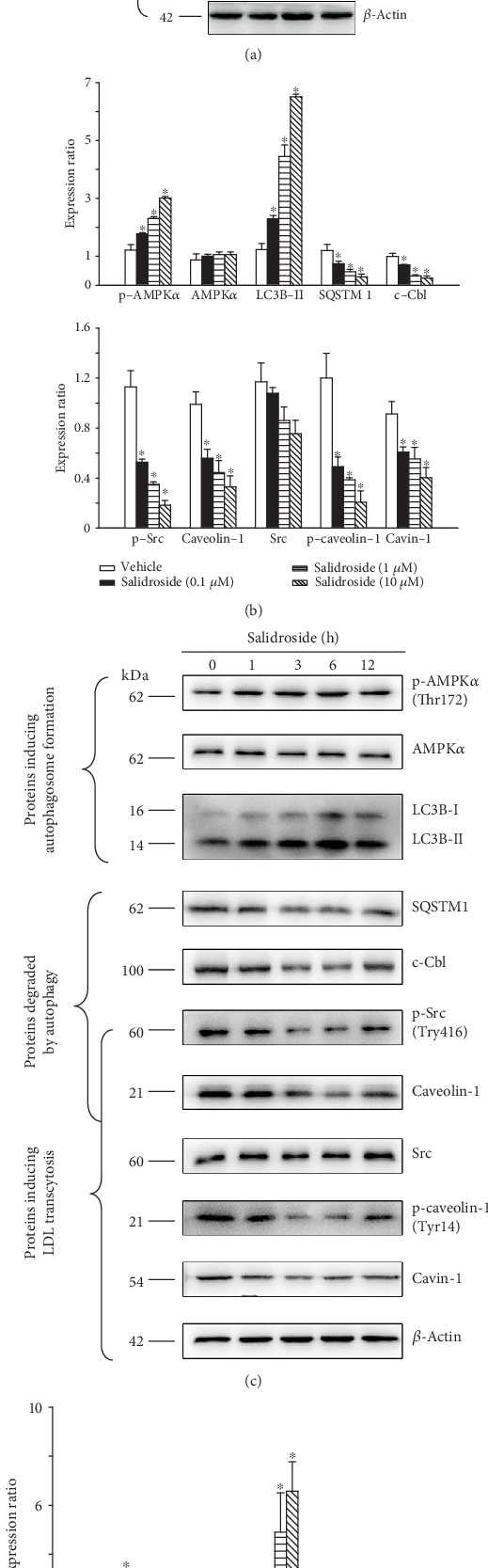
Salidroside activates AMPK and stimulates autophagic degradation of p-Src and caveolin-1 in a dose-dependent manner. (a, b) HUVECs were treated with indicated concentration of salidroside for 3 h, and then, cells were lysed, followed by whole-cell protein extraction. Whole-cell lysates from HUVECs were subjected to western blots analyses for indicated proteins, with *β*-actin as a loading control. Representative western blotting analyses of the indicated proteins (a) and summary bar graph showing the expression of the indicated proteins (b). (c, d) HUVECs were treated with salidroside (1 *μ*M) for the indicated time, and then, cells were lysed, followed by whole-cell protein extraction. Whole-cell lysates from HUVECs were subjected to western blot analyses for indicated proteins, with *β*-actin as a loading control. Representative western blotting analyses of the indicated proteins (c) and summary bar graph showing the expression of the indicated proteins (d). *n* = 3, ^∗^*p* < 0.05 versus vehicle control group.

The AMPK signaling pathway is a pathway to induce formation of the phagophore, a crescent-shaped double membrane that expands and fuses to form a double-membrane vesicle known as the autophagosome [30–32]. Therefore, we next evaluated the treatment effect of salidroside on AMPK activation. As depicted in Figures 3(a) and 3(b), AMPK phosphorylation was significantly upregulated by salidroside treatment in a concentration-dependent manner (0.1 *μ*M, 1 *μ*M, and 10 *μ*M),which was consistent with the findings presented in our previous study in which we showed that salidroside activated AMPK by increasing the ratio of AMP : ATP [19–21, 33]. In general, the covalent conjugation of a soluble form of LC3B (LC3B-I) with phosphatidylethanolamine to form a nonsoluble form (LC3B-II) is a major hallmark of autophagy [34]. We monitored changes in autophagy by analyzing the abundance of LC3B-II and the classical autophagic substrate SQSTM1 (sequestosome 1) as well as c Casitas B-cell lymphoma (c-Cbl) [35]. As shown in Figures 3(a) and 3(b), salidroside treatment resulted in a significant increase in LC3B-II and attenuated accumulation of the autophagy substrates SQSTM1 and c-Cbl. Moreover, active Src (as represented by p-Src) has been shown to be a target for the autophagosome, which was subsequently delivered to the lysosome for degradation through interaction with c-Cbl [36]. As illustrated in Figures 3(a) and 3(b), accumulation of p-Src was decreased by salidroside treatment, which was consistent with a downregulated expression of c-Cbl. In our previous study, we demonstrated that caveolin-1 was recruited to autophagosome for autolysosome degradation by direct interaction with LC3B-II [17]. Moreover, downregulation of caveolin-1 may lead to a loss in cavin-1 stability [37–39]. As depicted in Figures 3(a) and 3(b), upon salidroside treatment, protein levels of the caveolae structure proteins (caveolin-1 and cavin-1) were inhibited, which was accompanied by a disrupted phosphorylation of caveolin-1. Additionally, salidroside treatment resulted in a significant increase in LC3B-II and attenuated accumulation of the autophagy substrates (SQSTM1, c-Cbl, p-Src, and caveolin-1) in a time-dependent manner, which was consistent with a downregulated expression of cavin-1 (Figures 3(c) and 3(d)). Taken together, treatment with salidroside may activate autophagy and thereby induce autophagic degradation of p-Src and caveolin-1.

### 3.4. Bafilomycin A_1_ Blocked Salidroside-Induced Autophagic Degradation of p-Src and Caveolin-1

**Figure 4:**
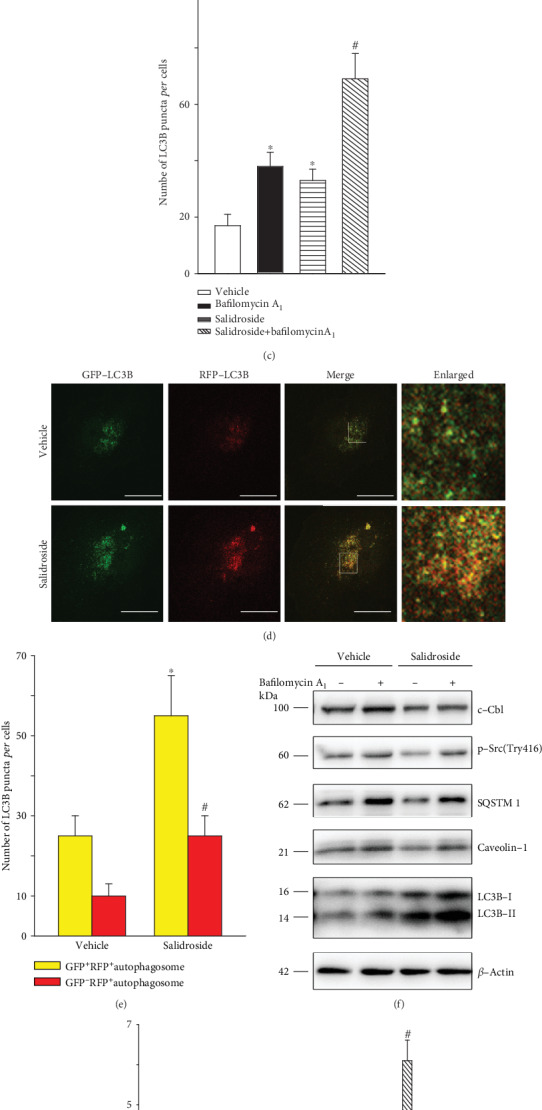
Bafilomycin A_1_ blocked salidroside-induced autophagic degradation of p-Src and caveolin-1. (a) HUVECs were exposed to salidroside (1 *μ*M) for 3 h and imaged by transmission electron microscopy. Representative images are shown. Arrow: autophagosomes or autolysosomes. Scale bar: 1 *μ*m. (b, c) HUVECs were transfected with GFP-LC3B plasmids for 24 h, followed by salidroside (1 *μ*M) treatment for 3 h in the presence or absence of bafilomycin A_1_ (100 nM, 2 h). GFP-LC3B puncta was visualized by confocal microscopy. (b) Representative fluorescent images are shown. Scale bars: 15 *μ*m. (c) The number of GFP-LC3B puncta in each cell was quantified, and at least 50 cells were included per group. ^∗^*p* < 0.05 versus vehicle; ^#^*p* < 0.05 versus bafilomycin A_1_ (*n* = 3). (d, e) HUVECs infected with GFP-RFP-LC3 lentivirus and subsequently subjected to salidroside stimulation (10 *μ*M, 3 h) and then observed for the change of both green and red fluorescence using a confocal microscope. (d) Representative fluorescent images are shown. Scale bar: 15 *μ*m. (e) The number of yellow or red-only LC3B puncta in each HUVECs in merged images was quantified, respectively, and at least 50 cells were included per group. ^∗^*p* < 0.05 versus yellow puncta in the vehicle group; ^#^*p* < 0.05 versus red-only puncta in the vehicle group (*n* = 3). (f, g) HUVECs were treated with salidroside (1 *μ*M) and bafilomycin A_1_ (100 nM) for 3 h. Whole-cell lysates from HUVECs were subjected to western blot analyses for indicated proteins, with *β*-actin as a loading control. Representative western blotting analyses of the indicated proteins (f) and summary bar graph showing the expression of the indicated proteins (g). ^∗^*p* < 0.05 versus vehicle; ^#^*p* < 0.05 versus salidroside (*n* = 3).

Our data using transmission electron microscopy showed an increase in autophagic vacuoles in the cytoplasm of HUVECs (Figure 4(a)). The increase of autophagosomes in cells is an intermediate process within the autophagic flux and reflects a balance between the rate of formation and degradation [40]. Thus, the increase in autophagosomes in salidroside-treated cells may be explained as follows: (1) salidroside increased the formation of autophagosomes, (2) salidroside prevented the fusion of lysosomes and autophagosomes, or (3) salidroside inhibited lysosome activity. To test these possibilities, bafilomycin A_1_, a lysosome inhibitor that can prevent fusion of lysosomes and autophagosomes and the degradation activity of lysosomes, was used. LC3B that was tagged at its N terminus using a fluorescent protein, such as GFP (GFP-LC3B), has previously been used to monitor autophagy through direct fluorescence microscopy that was measured as an increase in punctate LC3B [40]. Therefore, overexpression of HUVECs with GFP-LC3B was performed by transfecting HUVECs with GFP-LC3B constructs. As shown in Figures 4(b) and 4(c), both salidroside and bafilomycin A_1_ significantly enhanced the appearance of LC3B punctate. Moreover, simultaneous treatment with salidroside and bafilomycin A_1_ further increased the number of LC3B punctate when compared with bafilomycin A_1_ treatment alone (Figures 4(b) and 4(c)). Subsequently, a tandem fluorescent-tagged GFP-RFP-LC3B construct was used to assess the roles of salidroside in autophagic flux. The GFP of this tandem autophagosome reporter is sensitive to pH and quenched in the acidic environment of the lysosome, whereas the RFP is resistant. Therefore, the fusion of autophagosomes with lysosomes results in the loss of yellow puncta and the appearance of red-only puncta. As shown in Figures 4(d) and 4(e), salidroside increased the numbers of both yellow puncta and red-only puncta. In addition, coincubation of salidroside with bafilomycin A_1_ significantly enhanced the abundance of LC3B-II; however, no significant effect was observed on the amount of SQSTM1 when compared to bafilomycin A_1_ treatment alone (Figures 4(f) and 4(g)). Interestingly, coincubation of salidroside and bafilomycin A_1_ restored accumulation of the autophagic substrate (c-Cbl, p-Src, SQSTM1, and caveolin-1), when compared to salidroside treatment alone (Figures 4(f) and 4(g)). Taken together, these findings demonstrated that salidroside disrupted the autophagic accumulation of p-Src and caveolin-1 through facilitating the formation of autophagosomes in endothelial cells.

### 3.5. Salidroside Increases Src-LC3B and Caveolin-1-LC3B Complexes

**Figure 5:**
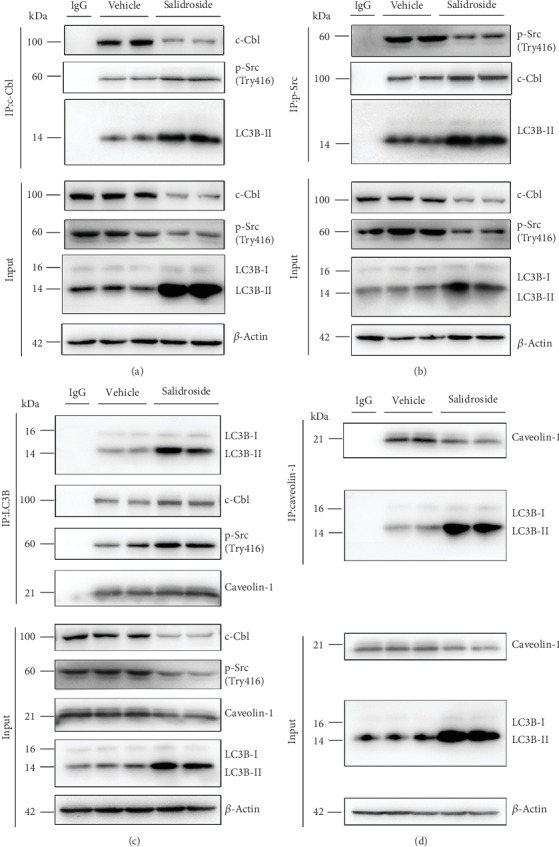
Salidroside increases the interaction between p-Src, c-Cbl, and LC3B, as well as the association between caveolin-1 and LC3B. (a–d) HUVECs were treated with salidroside (1 *μ*M) for 3 h. Whole-cell lysates from HUVECs were immunoprecipitated with c-Cbl antibody (a; IP: c-Cbl), p-Src antibody (b; IP: p-Src), LC3B antibody (c; IP: LC3B), or caveolin-1 antibody (d; IP: caveolin-1). Cellular *β*-actin from the same samples for IP served as a loading control (*n* = 3).

During autophagy, autophagic cargo is recruited to the autophagosome by interacting with the autophagosomal marker protein LC3B [35]. Interaction with the Src-LC3B complex is mediated by c-Cbl. Therefore, another pathway to demonstrate that salidroside promoted the autophagic degradation of p-Src and caveolin-1 was to investigate the interaction between p-Src, caveolin-1, and LC3B by coimmunoprecipitation experiments. As shown in Figures 5(a) and 5(b), the association between p-Src and c-Cbl and LC3B-II in HUVECs was significantly increased by salidroside treatment. Furthermore, salidroside treatment significantly increased the binding of caveolin-1 and LC3B-II (Figures 5(c) and 5(d)). Thus, these findings further demonstrated that salidroside accelerated the autophagic degradation of p-Src and caveolin-1.

### 3.6. Compound C, an AMPK Inhibitor, Prevented Salidroside-Induced Autophagic Degradation of p-Src and Caveolin-1

**Figure 6:**
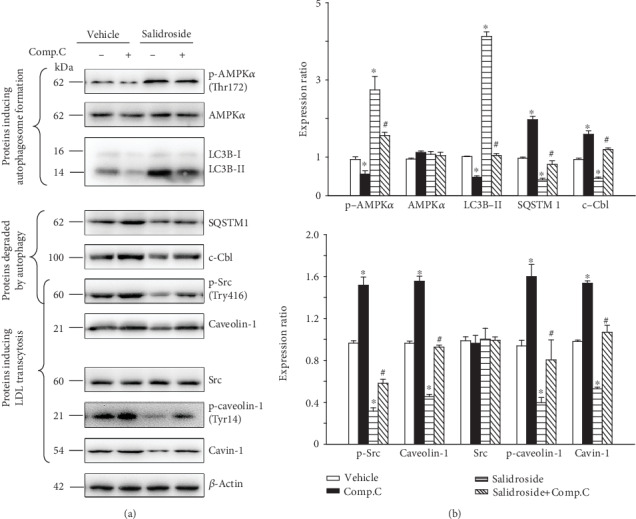
Compound c prevents salidroside-induced autophagic degradation of p-Src and caveolin-1. HUVECs were treated with indicated concentration of salidroside (1 *μ*M) for 3 h in the presence or absence of compound c (10 *μ*M, 3 h). Whole-cell lysates from HUVECs were subjected to western blot analyses for indicated proteins, with *β*-actin as a loading control. Representative western blotting analyses of the indicated proteins (a) and summary bar graph showing the expression of the indicated proteins (b). *n* = 3, ^∗^*p* < 0.05 versus vehicle. ^#^*p* < 0.05 versus salidroside (*n* = 3).

In this study, we also investigated whether salidroside induced the autophagic degradation of p-Src and caveolin-1 by activating AMPK. As presented in Figures 6(a) and 6(b), coincubation of salidroside and compound c attenuated the level of p-AMPK, when compared to salidroside treatment alone. In addition, the abundance of LC3B-II as induced by salidroside treatment was also decreased by administration of compound c. Conversely, simultaneous treatment of salidroside and compound c restored salidroside-induced autophagic degradation of SQSTM1, c-Cbl, p-Src, and caveolin-1 (Figures 6(a) and 6(b)). As a result, the restored expression of caveolin-1 enhanced the stability of cavin-1 (Figures 6(a) and 6(b)). Collectively, salidroside treatment promoted the autophagic degradation of p-Src and caveolin-1 by activating AMPK.

### 3.7. Salidroside Alleviates Atherosclerotic Lesion Formation in ApoE^−/−^ Mice

**Figure 7:**
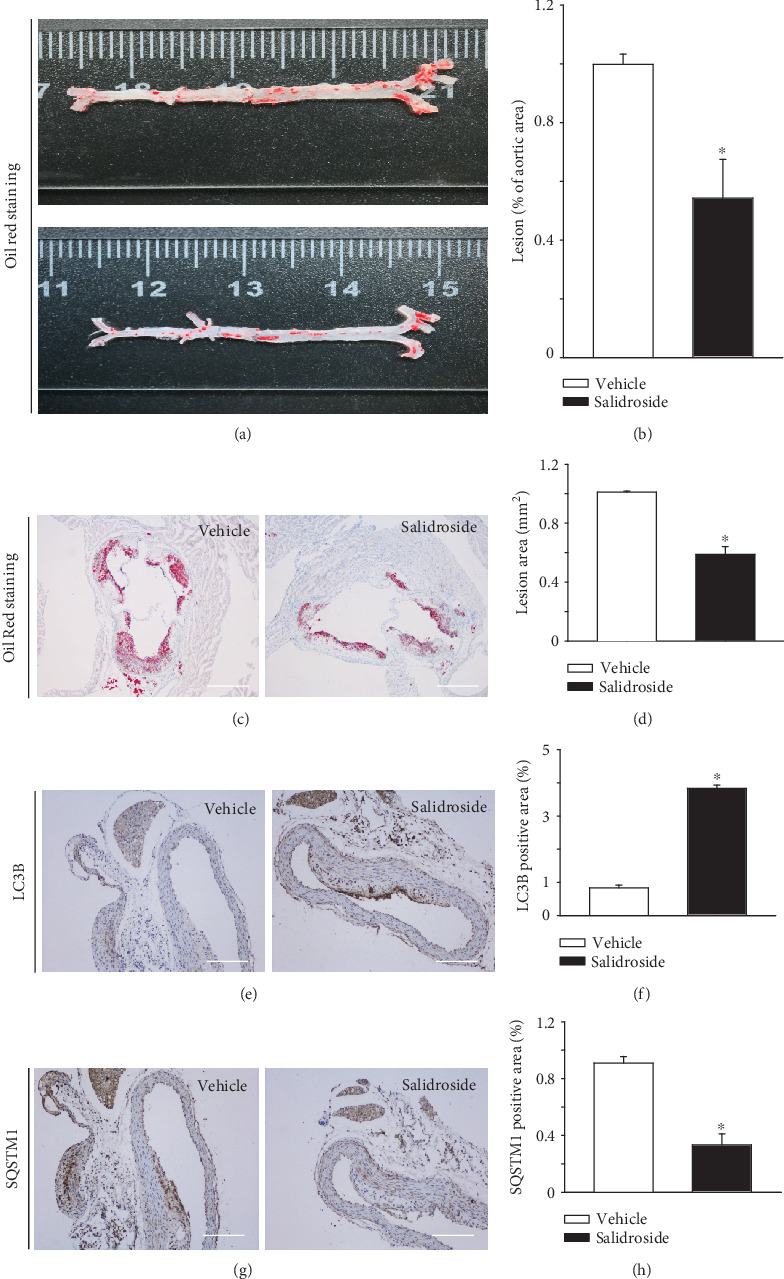
Salidroside alleviates atherosclerotic lesion formation in ApoE^−/−^ mice. 14-week-old male ApoE^−/−^ mice were randomly assigned to the vehicle group and the salidroside group. The vehicle group received p.o. with 0.9% saline (*n* = 7), and the salidroside group received p.o. with salidroside 50 mg·kg^−1^·day^−1^; *n* = 7) for 8 weeks and then killed under anesthesia. (a, b) Histological analyses of the atherosclerotic lesion area (Oil Red O staining) in the ascending aorta. (a) Representative images of the Oil Red O-stained aorta. (b) Quantitative summary of the percentage of the area of atherosclerotic lesion in the ascending aorta. (c, d) Histological analyses of the atherosclerotic lesion area (Oil Red O staining) in the root of the aorta indicated group. (c) Representative images of Oil Red O-stained aortic root sections. Scale bars = 600 *μ*m. (d) Quantitative summary of the percentage of the area of atherosclerotic lesion in the aortic root. (e–h) Immunostaining for LC3B (e) or SQSTM1 (g) in aortic root sections. Scale bars = 500 *μ*m. Quantitative summary of the expression of LC3B (f) and SQSTM1 (h) in aortic root endothelium.

Compared with the vehicle group, the relative surface area of the atherosclerotic lesion was markedly smaller in the salidroside (50 mg/kg) group (Figures 7(a) and 7(b). Furthermore, salidroside significantly delayed the formation of atherosclerotic lesions, characterized by the lipid deposition (Oil Red O staining positive) in the subendothelial space (Figures 7(c) and 7(d)). To determine the effect of salidroside on the autophagy level in ApoE^−/−^ mice, immunohistochemistry was conducted to measure the expression of LC3B and SQSMT1 in the aortic plaques. As shown in Figures 7(e) and 7(h), a significant increase in LC3B and an attenuated accumulation of the autophagy substrate SQSTM1 were observed in salidroside (50 mg/kg)-treated mice. All of these results demonstrated that salidroside delayed the formation of atherosclerotic lesions, by activating autophagy.

## 4. Discussion

The retention of LDL particles in the subendothelial space following LDL transcytosis across endothelial cells initiates the development of atherosclerosis [5, 23, 41]. In previous studies, it has been shown that autophagy is an emerging therapeutic target for preventing the development and progression of atherosclerosis [13, 14, 16, 42]. Thus, targeting the crossed pathway to activate autophagy and interrupt LDL transcytosis may be a promising antiatherosclerotic strategy. In the present study, we demonstrated that salidroside treatment decreased LDL transcytosis across endothelial cells by inducing the autophagic degradation of active Src and caveolin-1.

Transcytosis of LDL particles across endothelial cells is predominantly mediated by caveolae, which are presented at a high frequency in endothelial cells (~10,000 *per* cells) [43]. In the process of LDL transcytosis across endothelial cells, LDL particles are first to be endocytosed by caveolae at the lumen side of the endothelial cell membrane, subsequently trafficked to the basolateral side, then exocytosed to the subendothelial space [44]. Overexpression of cavin-1 and caveolin-1 increased the number of caveolae in the cell membrane, which upregulated LDL transcytosis [41]. The phosphorylation of caveolin-1 on tyrosine 14 triggers caveolae internalization from the plasma membrane and thereby accelerates caveolae-mediated LDL endocytosis [10]. Furthermore, phosphorylation of caveolin-1 on tyrosine 14 induced caveolae formation by enhancing caveolin-1 and cavin-1 transcription [11].

Activating AMPK induced formation of the phagophore or isolation membrane, a crescent-shaped double membrane that expands and fuses to form a double-membrane vesicle, the autophagosome [14]. In our previous study, we demonstrated that salidroside treatment moderately depolarized Δ*ψm* and increased the cellular AMP : ATP ratio, which ultimately activated AMPK [19, 20].

In an established *in vitro* model of LDL transcytosis across the endothelial cell monolayer, we first documented that salidroside interfered with LDL transcytosis that was restored by the AMPK inhibitor, compound c and *AMPKα* siRNA. Overexpression of exogenous p-caveolin-1 in salidroside-treated HUVECs by the transfection of the cells with a Y14D construct (phosphomimicking caveolin-1 on tyrosine 14) significantly reversed salidroside-suppressed LDL transcytosis.

An intermediate event of LDL transcytosis across endothelial cells is that LDL particles are endocytosed into cells but still need to be excreted out the other side of the cells. Therefore, the concentration of LDL within the cells reflects the amount of LDL transcytosis. Indeed, observations in these two sets of experiments were consistent with the data obtained in the *in vitro* transcytosis model as mentioned above. The transfection of *AMPKα* siRNA, *ATG5* siRNA, and Y14D constructs prevented the downregulation of LDL uptake upon salidroside treatment. Thus, these data provided compelling evidence, showing that salidroside inhibited LDL transcytosis.

Next, the treatment effect of salidroside on autophagy was elucidated. In our study, we determined that treatment with salidroside enhanced the phosphorylation of AMPK on Thr172 and increased the abundance of LC3B-II in a dose-dependent manner as well as in a time-dependent manner. Conversely, accumulation of autophagic substrates, including SQSTM1, c-Cbl, p-Src, and caveolin-1, was significantly disrupted by salidroside treatment. As a result, p-caveolin-1 expression was attenuated. The lost stability of caveolin-1 may lead to increased degradation of cavin-1. This phenomenon was also observed in our study.

Furthermore, transmission electron microscopy was performed to further ascertain that salidroside treatment activated autophagy. We found that treatment with salidroside significantly increased autophagic vacuoles in the cytoplasm. Moreover, bafilomycin A_1_, a type of drug that is used to prevent the fusion of lysosomes and autophagosomes and the degradation activity of lysosomes [34], further increased the number of LC3B punctate in salidroside-treated cells. Furthermore, the autophagic degradation of caveolin-1, p-Src, c-Cbl, and SQSTM1 was blocked by bafilomycin A_1_. Taken together, these results strongly suggested that salidroside activated autophagy.

In our previous study, we demonstrated that caveolin-1 was recruited to the autophagosome for autophagic degradation by directly interacting with LC3B-II [17]. Interestingly, active Src (indicated by autophosphorylation of Src on Tyr 416), which induced the phosphorylation of caveolin-1 on tyrosine 14, was also found to be a target of autophagosome for degradation by their association with autophagy cargo, c-Cbl [36]. Therefore, we next examined whether treatment with salidroside enhanced the interaction between c-Cbl, p-Src, and LC3B. We observed that salidroside strongly enhanced the association of c-Cbl, p-Src, and LC3B. In addition, binding of caveolin-1 and LC3B was also significantly increased. Thus, these data indicated that treatment with salidroside stimulated the autophagic degradation of p-Src and caveolin-1.

Activation of AMPK directly initiates the formation of autophagosomes. Moreover, the expression of p-AMPK, indicating the activation of AMPK, is increased upon salidroside stimulation. To further elucidate the role of AMPK in salidroside-induced autophagic degradation of p-Src and caveolin-1, in this study, compound c, which suppresses AMPK activation, was used. Our data showed that administration of compound c markedly attenuated the activation of salidroside-stimulated AMPK. Moreover, the increased amount of LC3B-IIas facilitated by salidroside, an autophagic marker, was also decreased by treatment with compound c. In addition, compound c restored the accumulation of SQSTM1, c-Cbl, p-Src, and caveolin-1 in salidroside-treated cells, which was consistent with the results of bafilomycin A_1_ treatment. Combined, these results strongly demonstrated that salidroside promoted autophagic degradation of p-Src and caveolin-1.

Consistent with our previous study [19], salidroside treatment significantly alleviated the development of atherosclerosis in ApoE^−/−^ mice. Meanwhile, consistent with the above *in vitro* findings, the expression of LC3B was increased, while the accumulation of SQSTM1 was attenuated in endothelium of atherosclerotic lesions. These observations further supported the *in vivo* significance of salidroside as strategies for the prevention or treatment of atherosclerosis.

**Figure 8:**
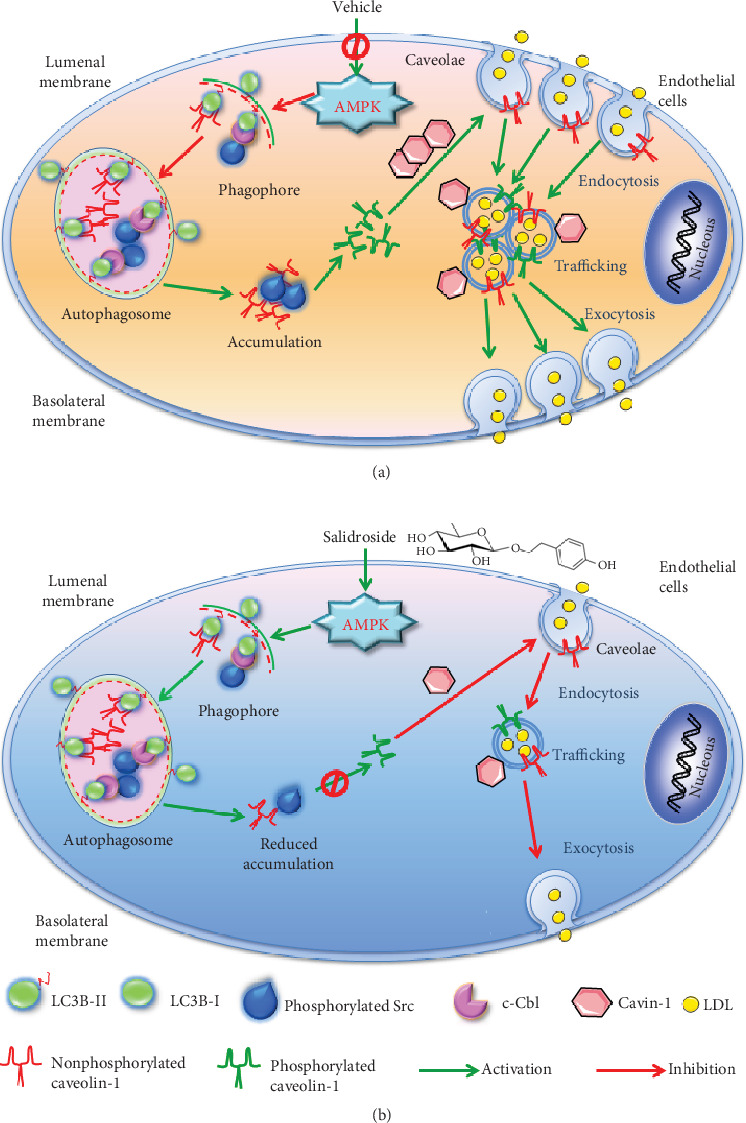
Proposed model of autophagy to regulate salidroside-suppressed LDL transcytosis: (a) vehicle-treated endothelial cells; (b) salidroside-treated endothelial cells.

In conclusion (Figure 8), upon salidroside stimulation, AMPK was activated, and subsequent formation of the autophagosome was promoted. As a result, increased amounts of p-Src and caveolin-1 are recruited to the autophagosome through interaction with LC3B-II, which in turn enhanced the autophagic degradation of p-Src and caveolin-1. Reduced accumulation of p-Src and caveolin-1 disrupted caveolin-1 phosphorylation on tyrosine 14, which ultimately inhibited caveolae-mediated LDL transcytosis across endothelial cells by decreasing the number of caveolae and by suppressing caveolae-mediated LDL endocytosis.

## Data Availability

The data used to support the findings of this study are available from the corresponding author upon request.

## Abbreviations

AMPK: Adenosine monophosphate-activated protein kinase
c-Cbl: c Casitas B-cell lymphoma
Comp.C: Compound c
DiI: 1,1′-Dioctadecyl-3,3,3′,3′-tetramethylindocar bocyanine perchlorate
FITC: Fluorescein isothiocyanate
HUVECs: Human umbilical vein endothelial cells
MAP1LC3/LC3: Microtubule-associated protein 1 light chain 3
LDL: Low-density lipoprotein
MFI: Mean fluorescence intensity
IP: Immunoprecipitation
p-AMPK: The phosphorylation of AMPK on threonine 14
p-caveolin-1: The phosphorylation of caveolin-1 on tyrosine 14
p-Src: The phosphorylation of Src on tyrosine 416
Src: SRC protooncogene, nonreceptor tyrosine kinase
SQSTM1/p62: Sequestosome 1
Y14D: Caveolin-1 mutant with the tyrosine (Y) 14 in caveolin-1 mutating into glycine (G)
Y14F: Caveolin-1 mutant with the tyrosine (Y) 14 in caveolin-1 mutating into phenylalanine (F).

## References

[1] Saeed A., Dabhadkar K., Virani S. S., Jones P. H., Ballantyne C. M., Nambi V. (2018). Cardiovascular disease prevention: training opportunities, the challenges, and future directions. *Current Atherosclerosis Reports*.

[2] Fogelstrand P., Boren J. (2012). Retention of atherogenic lipoproteins in the artery wall and its role in atherogenesis. *Nutrition, Metabolism, and Cardiovascular Diseases*.

[3] Frank P. G., Pavlides S., Lisanti M. P. (2009). Caveolae and transcytosis in endothelial cells: role in atherosclerosis. *Cell and Tissue Research*.

[4] Quest A. F., Leyton L., Parraga M. (2004). Caveolins, caveolae, and lipid rafts in cellular transport, signaling, and disease. *Biochemistry and Cell Biology*.

[5] Frank P. G., Woodman S. E., Park D. S., Lisanti M. P. (2003). Caveolin, caveolae, and endothelial cell function. *Arteriosclerosis, Thrombosis, and Vascular Biology*.

[6] Frank P. G., Lisanti M. P. (2004). Caveolin-1 and caveolae in atherosclerosis: differential roles in fatty streak formation and neointimal hyperplasia. *Current Opinion in Lipidology*.

[7] Razani B., Woodman S. E., Lisanti M. P. (2002). Caveolae: from cell biology to animal physiology. *Pharmacological Reviews*.

[8] Cao H., Courchesne W. E., Mastick C. C. (2002). A phosphotyrosine-dependent protein interaction screen reveals a role for phosphorylation of caveolin-1 on tyrosine 14: recruitment of C-terminal Src kinase. *The Journal of Biological Chemistry*.

[9] Lee H., Volonte’ D., Galbiati F. (2000). Constitutive and growth factor-regulated phosphorylation of caveolin-1 occurs at the same site (Tyr-14) in vivo: identification of a c-Src/Cav-1/Grb7 signaling cassette. *Molecular Endocrinology*.

[10] del Pozo M. A., Balasubramanian N., Alderson N. B. (2005). Phospho-caveolin-1 mediates integrin-regulated membrane domain internalization. *Nature Cell Biology*.

[11] Joshi B., Bastiani M., Strugnell S. S., Boscher C., Parton R. G., Nabi I. R. (2013). Phosphocaveolin-1 is a mechanotransducer that induces caveola biogenesis via Egr1 transcriptional regulation. *Journal of Cell Biology*.

[12] Axe E. L., Walker S. A., Manifava M. (2008). Autophagosome formation from membrane compartments enriched in phosphatidylinositol 3-phosphate and dynamically connected to the endoplasmic reticulum. *The Journal of Cell Biology*.

[13] Martinet W., de Meyer G. R. Y. (2008). Autophagy in atherosclerosis. *Current Atherosclerosis Reports*.

[14] Choi A. M. K., Ryter S. W., Levine B. (2013). Autophagy in human health and disease. *New England Journal of Medicine*.

[15] Ouimet M. (2013). Autophagy in obesity and atherosclerosis: interrelationships between cholesterol homeostasis, lipoprotein metabolism and autophagy in macrophages and other systems. *Biochimica et Biophysica Acta*.

[16] De Meyer G. R. Y., Grootaert M. O. J., Michiels C. F., Kurdi A., Schrijvers D. M., Martinet W. (2015). Autophagy in vascular disease. *Circulation Research*.

[17] Bai X., Yang X., Jia X. (2020). CAV1-CAVIN1-LC3B-mediated autophagy regulates high glucose-stimulated LDL transcytosis. *Autophagy*.

[18] Bai X. L., Deng X. L., Wu G. J., Li W. J., Jin S. (2019). Rhodiola and salidroside in the treatment of metabolic disorders. *Mini Reviews in Medicinal Chemistry*.

[19] Xing S. S., Yang X. Y., Zheng T. (2015). Salidroside improves endothelial function and alleviates atherosclerosis by activating a mitochondria-related AMPK/PI3K/Akt/eNOS pathway. *Vascular Pharmacology*.

[20] Zheng T., Yang X., Wu D. (2015). Salidroside ameliorates insulin resistance through activation of a mitochondria-associated AMPK/PI3K/Akt/GSK3*β* pathway. *British Journal of Pharmacology*.

[21] Wu D., Yang X., Zheng T. (2016). A novel mechanism of action for salidroside to alleviate diabetic albuminuria: effects on albumin transcytosis across glomerular endothelial cells. *American Journal of Physiology-Endocrinology and Metabolism*.

[22] Ganguly A., Zhang H., Sharma R., Parsons S., Patel K. D. (2012). Isolation of human umbilical vein endothelial cells and their use in the study of neutrophil transmigration under flow conditions. *Journal of Visualized Experiments*.

[23] Bian F., Yang X., Zhou F. (2014). C-reactive protein promotes atherosclerosis by increasing LDL transcytosis across endothelial cells. *British Journal of Pharmacology*.

[24] Cankova Z., Huang J. D., Kruth H. S., Johnson M. (2011). Passage of low-density lipoproteins through Bruch’s membrane and choroid. *Experimental Eye Research*.

[25] Kilkenny C., Browne W., Cuthill I. C., Emerson M., Altman D. G., NC3Rs Reporting Guidelines Working Group (2010). Animal research: reporting in vivo experiments: the ARRIVE guidelines. *British Journal of Pharmacology*.

[26] Matsubara J., Sugiyama S., Sugamura K. (2012). A dipeptidyl peptidase-4 inhibitor, des-fluoro-sitagliptin, improves endothelial function and reduces atherosclerotic lesion formation in apolipoprotein E-deficient mice. *Journal of the American College of Cardiology*.

[27] Missiou A., Köstlin N., Varo N. (2010). Tumor necrosis factor receptor-associated factor 1 (TRAF1) deficiency attenuates atherosclerosis in mice by impairing monocyte recruitment to the vessel wall. *Circulation*.

[28] Schwedler S. B., Amann K., Wernicke K. (2005). Native C-reactive protein increases whereas modified C-reactive protein reduces atherosclerosis in apolipoprotein E-knockout mice. *Circulation*.

[29] Li W., Yang X., Xing S. (2014). Endogenous ceramide contributes to the transcytosis of oxLDL across endothelial cells and promotes its subendothelial retention in vascular wall. *Oxidative Medicine and Cellular Longevity*.

[30] Egan D. F., Shackelford D. B., Mihaylova M. M. (2011). Phosphorylation of ULK1 (hATG1) by AMP-activated protein kinase connects energy sensing to mitophagy. *Science*.

[31] Kim J., Kim Y. C., Fang C. (2013). Differential regulation of distinct Vps34 complexes by AMPK in nutrient stress and autophagy. *Cell*.

[32] Kim J., Kundu M., Viollet B., Guan K. L. (2011). AMPK and mTOR regulate autophagy through direct phosphorylation of Ulk1. *Nature Cell Biology*.

[33] Xing S., Yang X., Li W. (2014). Salidroside Stimulates Mitochondrial Biogenesis and Protects against H2O2-Induced Endothelial Dysfunction. *Oxidative Medicine and Cellular Longevity*.

[34] Mizushima N., Yoshimori T. (2014). How to interpret LC3 immunoblotting. *Autophagy*.

[35] Khaminets A., Behl C., Dikic I. (2016). Ubiquitin-dependent and independent signals in selective autophagy. *Trends in Cell Biology*.

[36] Sandilands E., Serrels B., McEwan D. (2011). Autophagic targeting of Src promotes cancer cell survival following reduced FAK signalling. *Nature Cell Biology*.

[37] Bosch M., Marí M., Herms A. (2011). Caveolin-1 deficiency causes cholesterol-dependent mitochondrial dysfunction and apoptotic susceptibility. *Current Biology*.

[38] Verma P., Ostermeyer-Fay A. G., Brown D. A. (2010). Caveolin-1 induces formation of membrane tubules that sense actomyosin tension and are inhibited by polymerase I and transcript release factor/cavin-1. *Molecular Biology of the Cell*.

[39] Aravamudan B., VanOosten S. K., Meuchel L. W. (2012). Caveolin-1 knockout mice exhibit airway hyperreactivity. *American Journal of Physiology. Lung Cellular and Molecular Physiology*.

[40] Klionsky D. J., Abdelmohsen K., Abe A. (2016). Guidelines for the use and interpretation of assays for monitoring autophagy (3rd edition). *Autophagy*.

[41] Zhang Y., Yang X., Bian F. (2014). TNF-*α* promotes early atherosclerosis by increasing transcytosis of LDL across endothelial cells: crosstalk between NF-*κ*B and PPAR-*γ*. *Journal of Molecular and Cellular Cardiology*.

[42] Vindis C. (2015). Autophagy: an emerging therapeutic target in vascular diseases. *British Journal of Pharmacology*.

[43] Simionescu M., Popov D., Sima A. (2009). Endothelial transcytosis in health and disease. *Cell and Tissue Research*.

[44] Bian F., Xiong B., Yang X., Jin S. (2016). Lipid rafts, ceramide and molecular transcytosis. *Frontiers in Bioscience*.

